# Profiling mouse cochlear cell maturation using 10× Genomics single-cell transcriptomics

**DOI:** 10.3389/fncel.2022.962106

**Published:** 2022-08-18

**Authors:** Zhenhang Xu, Shu Tu, Caroline Pass, Yan Zhang, Huizhan Liu, Jack Diers, Yusi Fu, David Z. Z. He, Jian Zuo

**Affiliations:** ^1^Department of Biomedical Sciences, Creighton University School of Medicine, Omaha, NE, United States; ^2^Lynch Comprehensive Cancer Research Center, Creighton University School of Medicine, Omaha, NE, United States

**Keywords:** scRNA-seq, transcriptome, cochlear cells, hair cell, cochlear maturation, lncRNA, C57B/L6 mouse

## Abstract

Juvenile and mature mouse cochleae contain various low-abundant, vulnerable sensory epithelial cells embedded in the calcified temporal bone, making it challenging to profile the dynamic transcriptome changes of these cells during maturation at the single-cell level. Here we performed the 10x Genomics single-cell RNA sequencing (scRNA-seq) of mouse cochleae at postnatal days 14 (P14) and 28. We attained the transcriptomes of multiple cell types, including hair cells, supporting cells, spiral ganglia, stria fibrocytes, and immune cells. Our hair cell scRNA-seq datasets are consistent with published transcripts from bulk RNA-seq. We also mapped known deafness genes to corresponding cochlear cell types. Importantly, pseudotime trajectory analysis revealed that inner hair cell maturation peaks at P14 while outer hair cells continue development until P28. We further identified and confirmed a long non-coding RNA gene *Miat* to be expressed during maturation in cochlear hair cells and spiral ganglia neurons, and *Pcp4* to be expressed during maturation in cochlear hair cells. Our transcriptomes of juvenile and mature mouse cochlear cells provide the sequel to those previously published at late embryonic and early postnatal ages and will be valuable resources to investigate cochlear maturation at the single-cell resolution.

## Introduction

The vertebrate inner ear comprises auditory and vestibular sensory end organs responsible for hearing and balance, respectively. The perception of sound is mediated by the sensory epithelium located in the cochlea. Previous studies have shed light on the development of cochlear epithelia at embryonic and early postnatal ages in mice (Kolla et al., 2020). However, cochlear development does not stop at postnatal day 7 (P7) but continues in mice after calcification of the temporal bone when hearing sensitivity improves gradually from P14 to P21 (Lim and Anniko, 1985). Additionally, many non-syndromic hearing losses are progressive during cochlear maturation (Zhang et al., 2020), thus highlighting the significance of understanding the molecular dynamics of cochlear maturation at the single-cell level.

In contrast to ∼100 million photoreceptors (i.e., rods and cones) in the retina, the mouse cochlea contains only ∼700 inner hair cells (IHCs) and ∼2,000 outer hair cells (OHCs) (Ehret and Frankenreiter, 1977), the two types of sensory hair cells (HCs). Moreover, these sensory epithelial cells are extremely vulnerable during isolation, especially considering they are embedded in a calcified temporal bone at juvenile and mature ages. These constraints pose a significant challenge in dissecting cochlear cells during the maturation processes at the molecular level.

Next-generation RNA-sequencing (RNA-seq) has been powerful to evaluate the expression dynamics of critical genes in various vulnerable-low-abundance cochlear cell types (Yan et al., 2013; Slatko et al., 2018). However, such studies are mainly based on dissociated pooled cochlear cells, such as IHCs, OHCs, supporting cells, spiral ganglion neurons (SGNs), and striatal cells (Scheffer et al., 2015; Tang et al., 2020). Fluorescence-activated cell sorting (FACS) or manual cell sorting is often performed to enrich and isolate individual cells based on fluorescence labeling or morphological characteristics (Rueda et al., 2019; West et al., 2022). Recently, transgenic RiboTag mice were successfully used to purify RNAs from specific cochlear cell types (Milon et al., 2021). The limitations of these enrichment approaches are that they usually require large numbers of input cells, well-characterized transgenic mouse drivers, and prolonged cell preparation (Paul and Huang, 2018).

In contrast, unbiased single-cell (sc)RNA-seq without utilizing FACS or manual sorting has been successfully performed on embryonic and early postnatal inner ears to elucidate the developmental transcriptomics of various cochlear epithelial cell types (Kolla et al., 2020). Such an approach can profile transcriptomes of all distinct cell types, further revealing heterogeneity within the same cell type, and allowing multi-time point trajectory analysis to dissect molecular dynamics of developmental processes of individual cells. However, only one study has reported scRNA-seq using the 10x Genomics platform after calcification of the temporal bone (P7-10 in mice) (Yamashita et al., 2018). Single nuclear (sn)RNA-seq using the 10x Genomics platform has been conducted in mature mouse cochleae (Korrapati et al., 2019; Renthal et al., 2019); however, this method, in theory, will not identify cytoplasmic transcripts (Grindberg et al., 2013).

Here we describe an efficient unbiased scRNA-seq experimental procedure using the 10x Genomics platform, applicable for the cochlear sensory epithelia from juvenile and mature mice. We successfully sequenced mouse cochlear cells at multiple ages after ossification of the temporal bone and obtained the transcriptome profiles of various cell types in the cochlea. Furthermore, we elucidated the transcriptomic changes of HCs during maturation from P7 to P28 and identified the dynamic expression pattern of one novel HC and SGN marker gene, *Miat*. Our results can be considered a sequel to similar studies of cochlear sensory epithelia from late embryonic and early postnatal mouse ages (Kolla et al., 2020).

## Materials and methods

### Animals

All C57BL/6J males and females were purchased from The Jackson Laboratory (Stock #000664). Breeding pairs were set up to obtain P14 and P28 mice. Same-sex litter mates were housed together in individually ventilated cages with 4–5 mice per cage in the Animal Resource Facility of Creighton University. All mice were maintained on a regular diurnal lighting cycle (12:12 light: dark) with ad libitum access to food and water. Environmental enrichment included nesting material and PVC pipe. The procedures were approved by the Institutional Animal Care and Use Committee at Creighton University. All mice were euthanized between 10 a.m to 12 p.m for the scRNA-seq experiment and RNA scope.

### Sample preparation and cochlear cell dissociation

**FIGURE 1:**
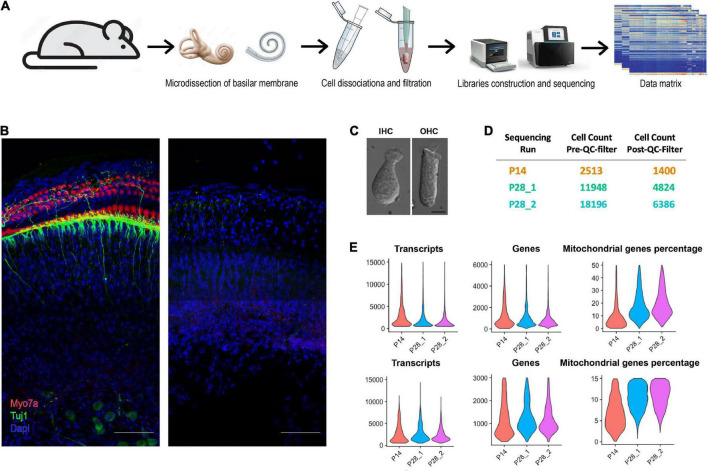
Workflow of ScRNA-seq analysis of cochlear cells. **(A)** Schematic diagram showing the procedure for cochlear cell scRNA-seq. **(B)** Isolated cochlear tissue before and after cell dissociation at P28. Scale bar = 50 μm. **(C)** Dissociated cochlear IHCs and OHCs at P28. Scale bar = 10 μm. **(D)** Cell number of each sequencing run before and after the QC filter. **(E)** Violin plots show the distribution of transcripts, genes, and the percentage of mitochondrial genes per cell for all scRNA-seq data.

Carbon dioxide euthanasia was performed on mice of all ages, and cochleae were dissected immediately afterwards and transferred into the iced DMEM/F12 (Gibco; #11320033) media in a 50 mm × 9 mm Petri Dish (Falcon; #351006). Forceps were used to remove the bony cochlear wall to expose the sensory epithelium entwining around the modiolus, hold the end of the coiled epithelial tissue and finally isolate the tissue gently. Part of the basal turn basilar membrane epithelial cells were either removed together with the bony cochlear wall or damaged by the forceps, so the epithelial cells we got were primarily from the upper basal (mid-cochlear) to apical turns. The cochlear tissue collected includes not only the whole sensory epithelial with the most critical component, the organ of Corti (OC), but also stria cells, Reissner’s membrane, SGNs, and other cell types (Figure 1B). Microdissection of 14 cochlear tissues from 7 mice was completed in less than 15 min from the time of euthanasia, and the cooperation of multiple personnel with experienced dissection skills was essential to minimize the dissection time. The tissues were then transferred into a single 1.5 ml tube using a trimmed 200 μl tip and incubated in 400 μl DMEM/F-12 media with 1 mg/ml Collagenase IV (Gibco; #17104019) for 10 min at room temperature (RT). At the end of incubation, collagenase was inactivated by adding an equal volume of DMEM/F-12 media with 10% fetal bovine serum (FBS). Tissues were then gently triturated with a trimmed 200 μl pipette to dissociate cells; after transferring half of the media to a new tube, tissues were then triturated with a new 200 μl pipette to further dissociate the tightly connected cells. All dissociated cells were passed through multiple 40 μm strainers, pelleted at 300 g for 5 min, and then extra media were removed to maintain only around 50 μl in final volume. Each sample preparation took less than one hour from euthanasia prior to the GEM generation and barcoding on the 10× Genomics Chromium Controller, and it was critical that tissues and cell solution were always kept on ice. A total of 7 and 14 mice were used for P14 and P28 scRNA-seq, respectively and two biological replicates (7 mice each) were used for P28 scRNA-seq.

### Library preparation and sequencing

Following the manufacturer’s recommendations, the emulsion droplets were constructed using a 10× Genomics Controller device. Library preparation was performed according to the instructions in the 10× Genomics Chromium Single Cell 3′ Reagent Kits V3.1. Quality control was performed for each individual cDNA library in an Agilent 2100 Bioanalyzer using a High Sensitivity DNA Kit (Agilent Technologies). All cDNA libraries were pooled for sequencing in an Illumina NextSeq 6000 sequencer aiming for 240 billion 150 bp long paired-end reads.

### Pre-processing of scRNA-seq data

Cell Ranger output data was processed using the Seurat package (version 4.0.4)^1^ in R software (version 4.1.0) for each scRNA-seq sample. Genes in at least three cells were included in the analysis; we filtered out the cells with numbers of expressed genes <200 or >3,000 and the cells with numbers of unique molecular identifiers (UMI) > 15,000. Cells with >15% mitochondrial genes were also excluded from the analysis.

### Data analysis

The Seurat objects were processed with the “Read10×” function, and the gene expression data from individual samples were converted into a natural logarithm and normalized under the same condition. The top 2,000 highly variable genes (HVGs) from the normalized expression matrix were identified for further principal component analysis (PCA). The integrated scRNA-seq data assay was created following the Seurat integration procedure. The clustering analysis was performed based on the individual data (P14) or integrated joint data (P7, P28). We then visualized the cell clusters on the 2D map produced with the t-SNE or UMAP method. Clusters were primarily annotated using SingleR^2^ for a reference-based scRNA-seq cluster annotation; differentially expressed genes (DEGs) with high discrimination abilities were then identified with the FindAllMarkers function. The cluster annotation correction was performed based on DEGs and the well-known cellular markers for cochlear cells (Table 1). For sub-clustering analysis of all HCs, similar procedures including variable genes identification, dimension reduction, and clustering identification were applied. The cluster-specific overrepresented Gene Set Enrichment Analysis (GSEA) biological process analysis was performed using the clusterProfiler package (version 4.0.5)^3^ based on the DEGs in the specific cell cluster compared to other remaining clusters in each dataset.

**TABLE 1:** Gene symbols for clustering annotation.

Cell type	Marker genes
OHC	*Myo6*, *Slc26a5*, Ocm (Self et al., 1999; Yu and Tang, 2008; Simmons et al., 2010)
IHC	*Myo6*, *Slc17a8*, *Otof* (Roux et al., 2006, 2009; Seal et al., 2008)
Spiral ganglion neuron	*Tubb3*, *Nefh* (Barclay et al., 2011; Locher et al., 2013)
Microglia	*Cx3cr1* (Jurga et al., 2020)
IPhC/IB/DC/PC/HeC	*Sox2* (Hume et al., 2007)
IPh/IB/Spiral ligament (SLg)	*Slc1a3* (Jin et al., 2003; Glowatzki et al., 2006; Udagawa et al., 2021)
Schwann cell	*Mpz*, *Mbp* (Kim et al., 2017)
Schwann cell (SC)/Satellite glial cell (SGC)	*Plp1* (Wan and Corfas, 2017)
Outer sulcus cell (OSC)/Inner sulcus cell (ISC)/DC/PC	*Gjb2* (Crispino et al., 2011)
Inner sulcus cell (ISC)/Hensen cell (HeC)/IPhc/IB	*Gata3* (Nishimura et al., 2017; Walters et al., 2017)
Reissner’s membrane (RM)	*Vmo1* (Peters et al., 2007)
Tympanic border cell (TBC)	*Emilin2* (Amma et al., 2003)
Spiral limbus (SLb) /Spiral ligament (SLg)	*Coch* (Robertson et al., 2001)
Marginal cell (MC)	*Kcnq1, Kcne1* (Tian and Johnson, 2020; Milon et al., 2021)
Intermediate cell (IC)	*Dct, Met* (Tian and Johnson, 2020; Milon et al., 2021)
Spindle cell/Root cell (SpC/RC)	*Slc26a4* (Milon et al., 2021)
Basal cell (BaC)	*Cldn11* (Milon et al., 2021)
Macrophage	*Cx3cr1* (Kaur et al., 2015; Rai et al., 2020)
B cell	*Cd79a* (Browne et al., 2003; Rai et al., 2020)
T cell	*Cd3g*, *Cd4* (Hong et al., 2011)
Granulocyte	*Itgam* (Fernekorn et al., 2007)

### Comparison of transcriptomes with previous publications

The adult HC bulk RNA-seq data from Li et al. (2018) were compared to our P28 HC scRNA-seq data, and the expression levels of all genes in both datasets were normalized using the same algorithm log1p which returns log (1+number). The expression levels of all shared genes were then mapped onto the scatter plots.

### Cellular localization of hearing loss genes

The hearing loss gene list was acquired from Kolla et al.’s (2020) previous publication to compare the cellular localization of the same genes from P7, P14 to P28. The cellular localization of hearing loss genes at P7 was directly acquired from Kolla et al.’s (2020) publication without any modification. The expression levels of all hearing loss genes at P14 and P28 were normalized based on z-score and then visualized in the heatmap. The analysis for each age was all done independently to avoid any interference among different datasets.

### Monocle trajectory analysis

Monocle2^4^ was used to perform the trajectory analyses and pseudotime heatmap. HCs were extracted from the preprocessed Seurat objects and then imported into the Monocle2. Only genes with mean expression ≥ 0.1 were used in the analysis. The reduceDimension function used the parameters method = “DDRTree” and max components = 3. Cell trajectory was captured using the orderCells function to arrange cells along the pseudotime. DEGs with the most predominant changes along with the pseudotime were calculated by comparing the transcriptome of HCs at one age to the other two ages. HC marker genes or DEGs at different ages were visualized with the plot_pseudotime_heatmap function; all genes were clustered into three subgroups based on their expression patterns.

### Localization of gene expression by RNAScope *in situ* hybridization and immunostaining

Cochleae from C57/BL6 mice of both sexes were collected and fixed in fresh 4% paraformaldehyde in PBS overnight at 4°C; samples were decalcified using 120 mM ethylenediaminetetraacetic acid (EDTA) for 2–3 days and pretreated according to the Advanced Cell Diagnostics (ACD) protocol for formalin-fixed paraffin-embedded tissue. *Miat* probe (#432521) and the RNAscope^®^ 2.5 HD Detection Reagents-RED (#322360) were ordered from ACD. Manufacture’s instruction and Liu et al.’s (2022) protocol were followed for the RNAscope. The following antibodies were used for immunostaining: Pcp4 (1:200, #HPA005792, Prestige Antibodies), goat anti-rabbit secondary antibody (1:800, #A-11011, Invitrogen). DAPI (40,6-diamidino-2-phenylindole) (1:1,000, #D1306, Invitrogen) was used to counterstain the nucleus. The samples were blocked at RT for 2 h in 0.2% Triton X-100 and 10% (v/v) heat-inactivated goat serum in PBS and then incubated with the primary antibodies overnight and corresponding secondary antibodies for 2 h at RT. All images were obtained on a Zeiss LSM 700 confocal microscope using the Z-stack with the same parameters.

## Result

### Implementation of the unbiased single-cell RNA seq experiment to P14 and P28 mouse cochleae

The hearing field has seen a rapid rise in scRNA-seq studies. In particular, unbiased scRNA-seq experiment has been successfully performed in neonatal mice before P7 prior to the calcification of the temporal bone (Kolla et al., 2020) and for P12, P26, and P33 mice with only a few endogenous HCs and SGNs sequenced (Yamashita et al., 2018). There is an urgent need to develop an available unbiased scRNA-seq experimental protocol for the calcified inner ear for our understanding of cochlear maturation at the single cell level.

We learned several crucial factors for successfully conducting the unbiased scRNA-seq experiments. First, minimizing the time of dissociating cochlear cells is essential for maintaining optimal cell viability and reducing the differences in dissociated cells across time points. The microdissection of the cochleae in our studies was performed by two experienced cochlear dissection experts. Second, instead of enzymatically lyzing the cochlear tissues with a strong enzyme (trypsin IV et al.) (Burns et al., 2015) or incubating the tissue with a set of mild enzymes for hours (Milon et al., 2021), we used collagenase IV for incubation of just 10 min at room temperature (RT), to loosen the connection among different cochlear cells. Tissues were then triturated using 200 μl tips, and trituration should be gentle for the first time and intense for the second time after removing half of the cell solution to a new tube. Most of the critical types of cochlear cells (including HCs and SGNs) are all located on the surface of the tissue; the cochlear tissue after the cell dissociation procedure in Figure 1B showed no Myosin7a or Tuj1 staining, indicating that the sensory epithelial cells were all dissociated. We examined the HCs 20 min after the dissociation under confocal microscopy, and all HCs showed normal morphology (Figure 1C). The cell solutions were used for the cDNA library construction 15 min after the cell dissociation.

We performed unbiased scRNA-seq at P14 and P28; there were two biological replicates at P28. For each sample, we used 7 mice and 14 ears. After the quality control (QC) filter, We obtained 16,909 sequenced genes at P14 and 19,054 sequenced genes for each biological replicate at P28. Transcriptomes of 1,400 cells were recovered at P14, and 4,824, 6,386 cells were recovered for the two biological replicates at P28 (Figure 1D). The number of transcripts, genes, and ratio of mitochondrial genes in each cell were all satisfactory (Figure 1E).

### Characterization of cell types in the juvenile and mature cochlea

**FIGURE 2:**
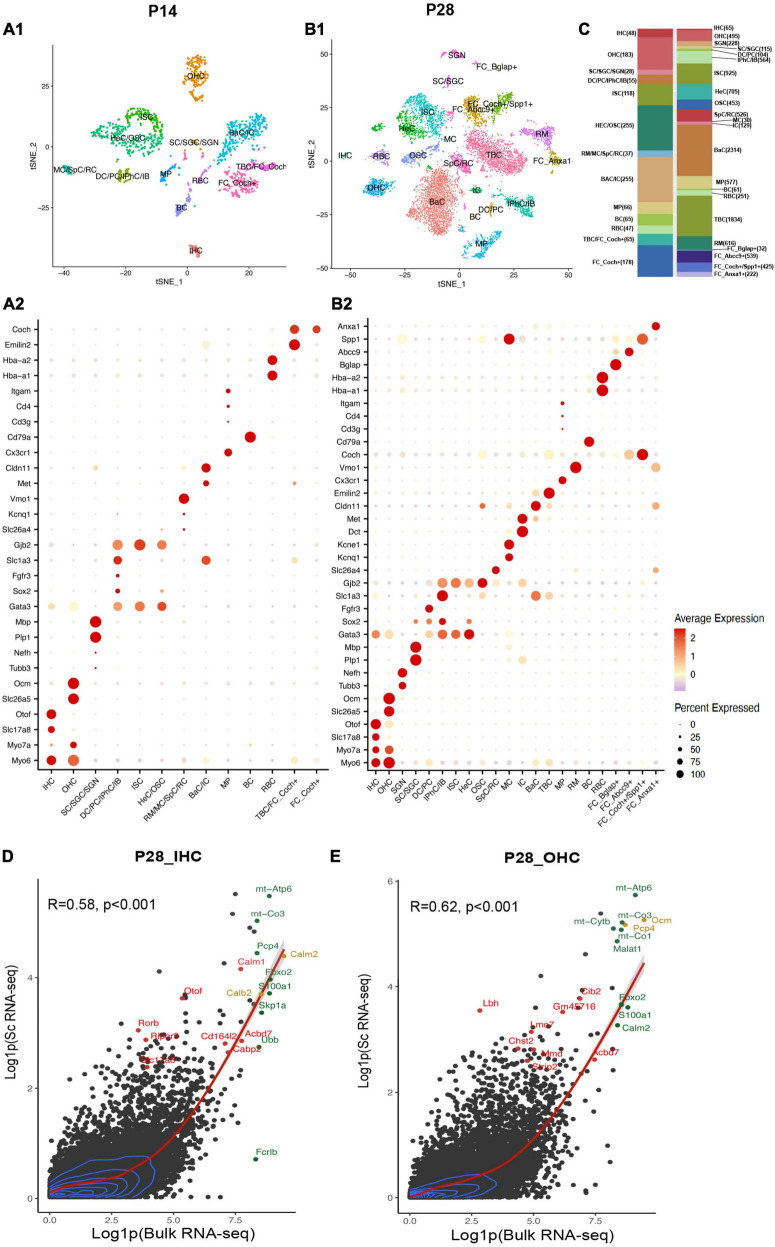
Single-cell transcriptional atlas of cochlear cells and comparison between scRNA-seq and bulk RNA-seq data. **(A1,B1)** tSNE plot of distinct cell types detected in C57/BL6 mice cochleae at P14 and P28. **(A2,B2)** Dot plot heatmap of average expression and cellular detection rate of representative canonical marker genes across different types of cells. **(C)** Relative proportion and numbers of cells of each cell type across two ages. Distinct cell types within the cochlea are color coded. **(D,E)** Scatter plot of the average expression level of IHC and OHC common genes in scRNA-seq and bulk RNA-seq data. The corrections of expression between two datasets are labeled with the red regression lines, and 2D density estimation was labeled with blue curves. The top 10 highly expressed genes in bulk RNA-seq data were labeled in green, the top 10 HC marker genes were labeled in red, and the overlapped genes were labeled in yellow. R, correlation coefficient. IHC, inner hair cell; OHC, outer hair cell; SGN, spiral ganglion neuron; SC, Schwann cell; SGC, satellite glial cell; DC, deiters’ cell; PC, pillar cell; IPhC, inner phalangeal cell; IB, inner border cell; ISC/OSC, inner/Outer sulcus cell; HeC, Hensen’s cell; SpC, spindle cell; RC, root cell; MC, marginal cell; IC, intermediate cell; BaC, basal cell; TBC, tympanic border cell; MP, macrophage; RM, Reissner’s membrane; BC, B cell; RBC, red blood cell; FC, fibroblast cell.

After filtering out low-quality cells, we used nearest-neighbor unsupervised clustering to identify and visualize cell populations using the t-distributed stochastic neighbor embedding (t-SNE) plots based on expression patterns (Supplementary Figures 1A,B). Clusters were annotated with SingleR.^5^ The SingleR annotation was further corrected based on the top differentially expressed genes (DEGs) for each cluster (Supplementary Data 1) and representative known marker genes for different cell types in the cochlea (Table 1). Cell types were identified (Figures 2A1,B1 and Supplementary Figures 1C,D), and the expression of representative canonical marker genes was shown (Figures 2A2,B2). We identified 48 IHCs and 183 OHCs at P14, 65 IHCs and 564 OHCs at P28, and 183 SGNs at P28, which provided a strong foundation for our downstream analysis of HCs. In addition, we identified other cell types of the cochlea: inner phalangeal cells (IPhCs), inner border cells (IBs), Deiters’ cells (DCs), pillar cells (PCs), Hensen’s cells (HeCs), inner and outer sulcus cells (ISCs, OSCs), Reissner’s membrane cells (RMs), tympanic border cells (TBCs) underneath the basilar membrane, macrophages (MPs), Schwann cells (SCs), satellite glial cells (SGCs), Spindle cells(SpCs), Root cells (RCs), Marginal cells (MCs), Basal cells (BaCs), Intermediate cells (ICs), B cells (BCs), and red blood cell (RBCs). We listed top marker genes for each cell type we identified (Supplementary Figures 1E,F). The cell-type compositions and numbers of cells for two ages were shown (Figure 2C).

### Comparison of our scRNA-seq data to published bulk RNA-seq data

Bulk RNA-seq with a large quantity of cells as input has been utilized broadly in the hearing field, among which HCs appear most difficult to obtain (Li et al., 2020). Li et al. (2018) have reported bulk RNA-seq data of manually sorted IHCs and OHCs from mice between P25 to P30. To allow for comparison of our scRNA-seq results with published bulk RNA-seq datasets, we aggregated and normalized the expression of all genes in HCs in our P28 unbiased scRNA-seq dataset and Li et al.’s (2018) bulk RNA-seq dataset from P25-30 mice.

For scRNA-seq data, the raw RNA counts were processed with the AverageExpression () and log1p () function, which allow us to get one average expression level of each gene in all 64 IHCs or 494 OHCs. Genes with average expression levels from this method were regarded as expressed in IHCs/OHCs (Supplementary Data 2). The bulk RNA-seq data were also processed with the log1p () function. 17,621 and 18,894 genes were detected in bulk RNA-seq data for IHCs and OHCs, respectively. Our scRNA-seq detected 11,132 and 15,007 genes for IHCs and OHCs, respectively. Bulk and scRNA-seq data both revealed OHCs to have more gene detection compared to IHCs. Most of the genes were expressed at a relatively low level in HCs for both datasets (Figures 2D,E). The expression levels of common genes in our scRNA-seq data correlated with those in the corresponding bulk RNA-seq data. The overall correlation coefficients between scRNA-seq and bulk RNA-seq datasets at P14 and P28 were 0.58 and 0.62, respectively. The top 10 highly expressed genes in the bulk RNA-seq data (Figures 2D,E, green dots) also showed high expression levels in scRNA-seq data. The top 10 HC marker genes in scRNA-seq data (Figures 2D,E, red dots) were also highly expressed in the corresponding bulk RNA-seq data. We observed in our scRNA-seq data that two of the top 10 IHC marker genes, *Calm2* and *Calb2*, and two of the top 10 OHC marker genes, *Ocm* and *Pcp4* (Figures 2D,E, yellow dots), were also among the top 10 highly expressed genes in bulk RNA-seq data. These comparisons confirmed that our scRNA-seq datasets are consistent with the previously reported bulk RNA-seq data and were able to identify HC marker genes.

### Expression levels and cellular localization of deafness genes

**FIGURE 3:**
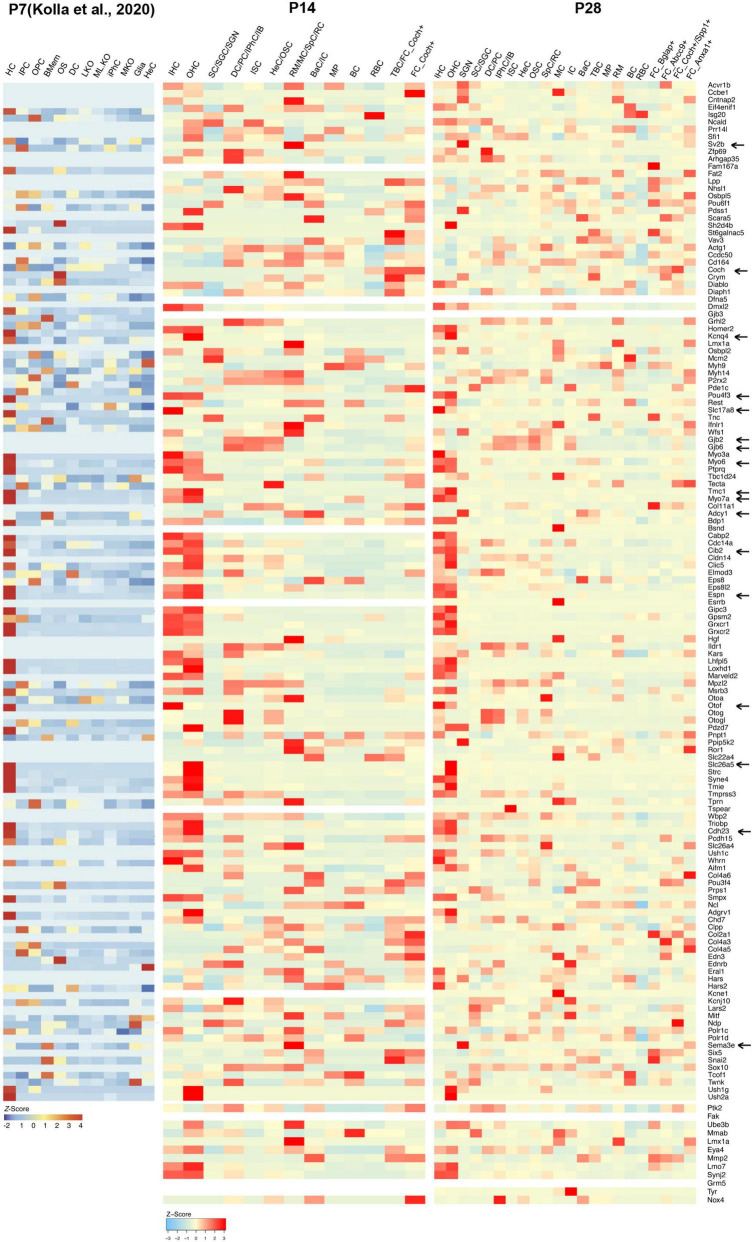
Cellular localization and expression levels of deafness genes from P7 to P28. Heatmaps showing the cell-type-specific expression (as a z-score for cell-type-averaged expression) for deafness genes in cochlear cells. Representative hearing loss genes discussed in the text were indicated by arrows.

Little is known about the expression levels of many known and potential deafness-related genes in the cochlea after the calcification of the temporal bone or the onset of hearing at juvenile and mature ages. To fill this gap, we calculated the average expression levels and cellular localization of deafness genes at P14 and P28 in our scRNA-seq datasets. The deafness genes were from previous publications (Liu et al., 2014, 2021; Kolla et al., 2020). We observed similar expression patterns of many deafness genes from P14 to P28 compared to what has been reported by Kolla et al. (2020) at P7 (Figure 3) and other reports (Angeli et al., 2012). Hair bundle development and functioning related genes *Myo6*, *Myo7a*, *Cdh23*, *Espn*, *Tmc1*, and *Cib2* were primarily expressed in IHCs and OHCs; synaptic transmission-related genes *Otof* and *Slc17a8* were primarily expressed in IHCs; *Slc26a5* and *Kcnq4* were primarily expressed in OHCs, and *Pou4f3* was highly expressed in IHCs and OHCs; gap junction related genes *Gjb2* and *Gjb6* were predominantly expressed in SCs and fibroblast cells; we also detected the expression of *Sema3e*, *Adcy1* and *Sv2b* in the SGNs. *Coch* was detected in the TBCs and fibroblast cells.

### Characterization of HC maturation in the cochlea

HCs are mechanosensitive cells in the cochlea and play a vital role in normal hearing function. There is an urgent need for a comprehensive understanding of HC transcriptomic changes after the onset of the hearing (∼P14) (Sonntag et al., 2011). We utilized the scRNA-seq dataset from Kolla et al. (2020) at P7 and compared it to our unbiased scRNA-seq datasets at P14 and P28. The time points of P7, P14, and P28 in mice represent the early postnatal development stage, the onset of hearing, and the maturation stage of HCs, respectively, enabling the study of HC maturation.

**FIGURE 4:**
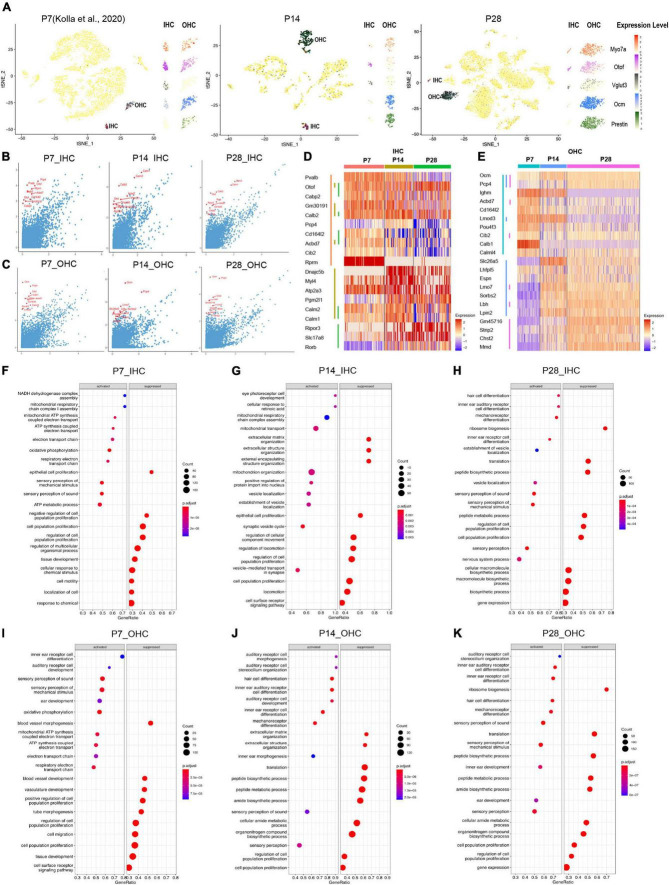
Single-cell transcriptomic analysis of HCs at P7, P14, and P28. **(A)** tSNE plot showing the overlayed and respective expression levels of the representative HC marker gene, Myo7a; IHC marker genes, Otof and Vglut3; OHC marker genes, Ocm, and Prestin. **(B,C)** The scatter plot showing the expression level of the DEGs in IHC and OHC (y-axis) by comparison with all other cochlear cells (x-axis). The top 10 HC marker genes in each comparison were labeled in red. **(D,E)** The heatmap of the expression levels of top 10 marker genes among IHC and OHC across different ages. HCs and their top 10 marker genes at three ages were labeled with distinct colored bars. **(F–K)** GSEA biological process terms enriched in HC-specific gene signatures. Activated and suppressed biological processes in IHC and OHC at P7, P14, and P28, respectively.

As shown in the tSNE plots and the high-resolution feature plots on the right of each tSNE plot (Figure 4A), canonical HC marker genes were only highly expressed in the IHC and/or OHC clusters. We identified marker genes for IHCs and OHCs at different ages by comparing the gene expression of IHCs/OHCs to other cell types, and the top 10 markers identified were labeled in red (Figures 4B,C). The differentially expressed genes (DEGs) between IHCs and OHCs were also identified (Supplementary Figure 2).

The IHC markers *Otof*, *Calb2*, and *Acbd7* are among the top 10 markers shared at all three ages; *Otof* and *Slc17a8*, increased expression gradually from P7 to P28 while *Rprm* maintained a high expression level at all three ages; other marker genes (*Pvalb*, *Cd16412*, *Acbd7*, *Cib2*) exhibited decreasing expression patterns from P7 to P28 (Figure 4D). Similar to IHCs, OHCs at different ages shared two common top 10 markers, *Ocm* and *Pcp4* (Figure 4E), while multiple unique top marker genes at P14 and P28, such as *Lmo7*, *Sorbs2*, *Lbh*, *Lpin2*, *Gm45716*, *Strip2* and *Chst2*, were also highly expressed in adult OHCs from the bulk RNA-seq analysis (Li et al., 2018). *Mmd* has an increased expression from P7 to P28 and most of the unique top markers for P7, such as *Lghm*, *Acbd7*, *Cd164l2*, *Lmod3*, *Pou4f3*, *Cib2*, *Calb1*, and *Calml4* showed a decreased expression in OHCs from P7 to P28. Interestingly, the motor protein Prestin coding gene *Slc26a5* had peak expression at P14, which indicates the maturation of mechanical contraction and elongation ability in OHCs occurred around the onset of hearing, consistent with previous electrophysiological studies (Hang et al., 2016).

We further investigated HC-specific gene signatures by performing Gene Set Enrichment Analysis (GSEA) (Figures 4F–K). The biological processes related to proliferation and cell migration were suppressed, and the biological processes related to inner ear differentiation and development were activated. There are more auditory system developmental biological processes presented at P14 in OHCs than in IHCs, indicating the OHCs were still maturing.

### Outer hair cells continue maturation from P7 to P28 while inner hair cell maturation peaks at P14

**FIGURE 5:**
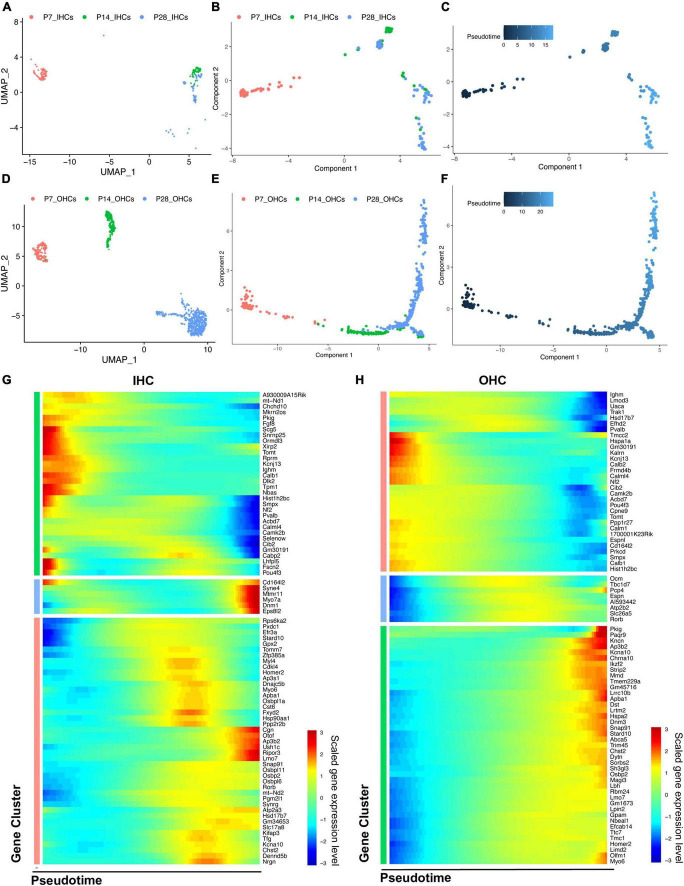
Trajectory analysis of cochlear HCs at P7, P14, and P28. **(A,D)** UMAP plots of the different HC populations at three ages. **(B,C,E,F)** Monocle 2 trajectory plots showing different types of IHC and OHC, and their pseudotime features. **(G,H)** The scaled expression level of combined top 50 marker genes at each age along the pseudotime for IHC and OHC.

Based on the distribution of the HC clusters from different ages (Figures 5A,D), we performed a trajectory analysis of all HCs at P7, P14, and P28 with Monocle 2 (Trapnell et al., 2014) to infer the HC maturation course (Figures 5B,E). The trajectory analysis indicated that IHCs and OHCs are clearly maturing after P7. We observed that IHCs have a more significant overlap between P14 and P28 than OHCs, indicating that the transcriptomic changes of IHCs dramatically slowed after P14, and the maturation of OHCs continued until P28 (Figures 5B,C,E,F).

We then combined the top 50 HC marker genes at each age and visualized their relative expression levels through pseudotime (Figures 5G,H). The expression levels of genes related to the early development of HCs, such as *Fgf8* and *Pou4f3*, were significantly reduced during maturation. Meanwhile, the genes related to the specific HC functions and survival, such as *Slc17a8*, *Otof*, *Ikzf2*, *Lbh*, and *Dnm3*, were gradually up-regulated along the trajectory maturation process. We also observed *Slc26a5*, *Ocm*, and *Espn* to have highest expression levels in the middle of the trajectory pseudotime.

Genes with the most predominant changes along with the pseudotime were visualized in Supplementary Figure 3. DEGs were acquired by comparing the transcriptomes of HCs between P14 and P7, P28 and P14, respectively, revealing two different maturation stages between P7 and P28. The GSEA biological processes related to the DEGs are shown in Supplementary Figure 4.

### Characterization of novel HC and SGN marker genes

**FIGURE 6:**
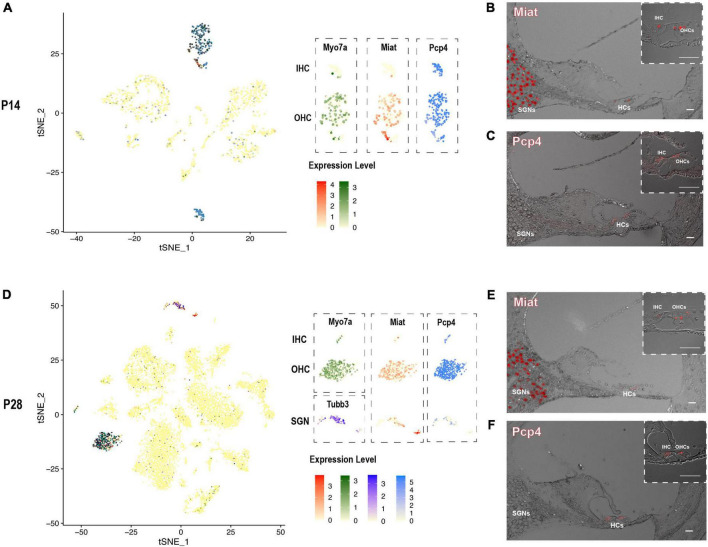
The expression pattern of newly identified HC and SGN marker gene *Miat* and *Pcp4*. **(A,D)** tSNE plot visualization of *Miat* expression in Myo7a highly expressed HCs and Tubb3 expressed SGNs at P14 and P28; tSNE plot visualization of *Pcp4* expression in HCs at P14 and P28. **(B,E)** In situ hybridization in P14 and P28 cochlear cross-sections showing the localization of the mRNA of *Miat* in HCs and SGNs. **(C,F)** Immunostaining in P14 and P28 cochlear cross-sections showing the expression of *Pcp4* in HCs. Scale bar = 20 μm.

After screening our HC and SGN maker gene list, there were two interesting genes *Miat* and *Pcp4* caught our eyes. The long non-coding RNA gene *Miat* has been reported to be functional in neuron development, myocardial infarction, schizophrenia, and malignant tumors (Da et al., 2020). Most recent studies showed that *Miat* is related to age-related hearing loss (Hao et al., 2019) and expressed in the cochleovestibular ganglion cells (Sun et al., 2022). Purkinje cell protein-4 (Pcp4) is a small IQ motif-containing protein that regulates the calmodulin-dependent signaling (Kim et al., 2014). Scheffer et al. have reported the expression of *Pcp4* in collected P7 hair cells utilizing FASC (Scheffer et al., 2015). We discovered *Miat* to be highly co-expressed with *Myo7a* and *Tubb3*, indicating that *Miat* was expressed in the HCs and SGNs. The expression level of *Miat* in the IHCs was relatively lower than in OHCs. *Pcp4* was highly expressed in HCs at P14 and P28 (Figures 6A,D).

We further utilized RNAscope to validate the expression of *Miat* in both HCs and SGNs at P14 and P28 (Figures 6B,E). The low expression of *Miat* in IHCs was detectable with a strong fluorescent signal at P14 while faint at P28. Immunostaining was used to validate the expression of *Pcp4* in HCs at P14 and P28 (Figures 6C,F). To further investigate the expression pattern of *Miat* and *Pcp4* during development, we used Kolla et al.’s (2020) embryonic and neonatal scRNA-seq data in the gEAR (Orvis et al., 2021) and found *Miat* was highly expressed in IHCs from E14 to P7, and its expression started in OHCs from E16 to P7 (Supplementary Figure 5); Pcp4 was highly expressed in HCs, prosensory cell, PCs, and DCs from E14 to P1, and its expression were concentrated in HCs at P7.

### Limitations of the study

Although we have presented scRNA-seq datasets for multiple ages after the calcification of the inner ear, there are still several limitations in our study. First, we only identified SGNs at P28, similar to previous embryonic and early postnatal datasets (Kolla et al., 2020). We think the number of SGNs we got from P14 is too minimal to be clustered. Second, cells in this dataset are primarily from the upper basal to apical turn, so the transcriptomics of HCs from the high frequency are likely not included. Third, more cells were sequenced at P28 compared to P14 and Kolla et al.’s (2020) P7 scRNA-seq data were from a different mouse strain (CD1), which may compromise the accuracy of the comparison. Fourth, compared to the plate-based Smart-seq full-length sequencing method, the droplet-based 10X Genomics chromium approach detected less genes, especially low abundance transcripts. Fifth, it would be ideal for providing *in vivo* functional data for *Miat* and *Pcp4*; however, it is beyond the scope of the current study.

## Discussion

Our study presents a comprehensive transcriptomic profiling of mouse cochlear cells using 10x Genomic scRNA-seq at juvenile and mature ages. Given the calcification of the temporal bone after P7–10 in mice, the ultra-low abundance and high vulnerability of cochlear cells upon isolation *in vitro*, it has been widely considered impractical to perform unbiased 10x Genomics scRNA-seq from juvenile and mature mouse inner ears. In fact, it was stated that P7 in mice is the age “as the best compromise between maturity and our ability to successfully dissociate and capture a significant number of cells” (Kolla et al., 2020). Based on our previous experience of conducting the unbiased scRNA-seq in mature cochleae (Yamashita et al., 2018), we perfect the whole procedure with more detailed protocols and bioinformatic analysis and successfully identify scRNA-seq profiles of distinct cochlear cells at P14 and P28. Specific parameters of our datasets are within acceptable ranges of published studies in hearing and other fields (Luecken and Theis, 2019). Bioinformatic analysis identifies cell types in the calcified cochlea, allowing us to uncover the transcriptome profiles of various cochlear cell types and further understand the complex maturation dynamics.

To further validate our results, we compared our datasets at P14 and P28 to previously published bulk RNA-seq data of HCs at similar ages (P15 and P25–30) and found that our datasets are consistent with those in bulk RNA-seq (Li et al., 2018). Given the difficulties of obtaining transcriptomes of extremely vulnerable HCs at these ages, we conjecture that scRNA-seq profiles of other cochlear cell types in our dataset should be consistent with those in other previous publications and faithfully recapitulate their dynamic maturation processes. Human fetuses develop hearing function around 27 weeks of age in utero (Litovsky, 2015) and can respond to sound stimuli at birth; however, mice initiate hearing around P14 (Sonntag et al., 2011) and develop mature hearing around P20 (Pujol et al., 1991). All known and potential deafness genes, including transcription factors and progressive deafness genes, are mapped here at cellular resolution in the complex cochlea at P14 and P28. Their cellular localizations are consistent with those in mouse cochlear P7 scRNA-seq data (Kolla et al., 2020). These analyses thus confirmed the reproducibility and accuracy of our scRNA-seq datasets which will be an excellent resource for studying human hearing loss-related genes after birth, ultimately shedding light on therapeutic targets in precision medicine.

Our bioinformatic analysis reveals not only significant similarities between the results at P7 (Kolla et al., 2020) and ours at P14-28 but also continuous transcriptomic changes from P7, P14 to P28 during cochlear maturation. HCs at these different ages (P7–28) share some common marker genes; however, many top marker genes change substantially, supporting that HCs are continuously maturing after P7. GSEA analyses reveal multiple biological processes essential for various auditory normal functions and deafness. Based on the Monocle 2 algorithms, we performed pseudotime trajectories of HCs that elucidate different maturation phases. The maturation of IHCs peaks at P14 while OHCs continue maturing until P28. Moreover, we present a collection of HC maturation genes, many of which have been studied before but not at relative levels over the trajectory timeline. Our analysis suggests that genes such as *Slc26a5* play essential roles in the specification of OHC function related to dynamic changes of stiffness at P14 and P28, while the gradually increasing expression of OHC marker genes *Ikzf2*, *Dnm3*, and *Lbh* further validates the specification of their functions. Similarly, *Otof*, *Lmo7*, and *Dnm1* are critical for the specification of IHC function along with the pseudotime.

Although the connection between the long non-coding RNA gene *Miat* and cochleovestibular ganglion cells has been made recently (Sun et al., 2022), nothing has been reported about *Miat* in the cochlea. *Pcp4* has been reported expressed in HCs but never identified as an HC marker. We discovered that *Miat* is a specific marker gene for HCs and SGNs, and *Pcp4* is a specific marker gene for HCs in our and previously published scRNA-seq data (Kolla et al., 2020). Their expression pattern is further validated by RNAscope *in situ* hybridization and Immunostaining at P14 and P28. These results suggest that *Miat* and *Pcp4* are potential fate-determination or differentiation genes for HCs and SGNs and are essential for maintaining their normal function.

In conclusion, we provide a practical and reproducible unbiased scRNA-seq experimental protocol and report reliable scRNA-seq profiles for various cochlear cell types in the mouse inner ear embedded in the calcified temporal bone at juvenile and mature ages. We further demonstrate the transcriptomic atlas of HCs during maturation and reveal novel marker genes, *Miat*, for HCs and SGNs; *Pcp4*, for HCs. Our datasets thus serve as valuable resources for studying the transcriptomes of various cell types in the juvenile and mature cochleae.

## Data availability statement

The datasets presented in this study can be found in online repositories. The name of the repository and accession number can be found below: National Center for Biotechnology Information (NCBI) Gene Expression Omnibus (GEO), https://www.ncbi.nlm.nih.gov/geo/, GSE202920. The Codes are available at Mendeley Data (https://data.mendeley.com/datasets/bbbjwps9b2).

## Ethics statement

The animal study was reviewed and approved by Institutional Animal Care and Use Committee at Creighton University.

## Author contributions

ZX and JZ designed experiments. ZX, ST, YZ, and HL performed the single cell RNA sequencing experiments. CP conducted the *in situ* hybridization experiments and edited the manuscript. JD conducted the immunostaining experiments. ZX performed the bioinformatic analysis, imaging, data analyses, and prepared figures. YF reviewed the design and bioinformatics analysis. DH reviewed the design of the experiment and edited the manuscript. ZX and JZ wrote the manuscript with edits from other authors. ST and CP contributed equally to this work and share second authorship. All authors contributed to the article and approved the submitted version.

## Footnotes

1
https://github.com/satijalab/seurat

2
https://bioconductor.org/packages/release/bioc/html/SingleR.html

3
https://github.com/YuLab-SMU/clusterProfiler

4
http://cole-trapnell-lab.github.io/monocle-release/

5
https://github.com/dviraran/SingleR

## Supplementary material

Supplementary Figure 1 **(A–D)** tSNE and UMAP plots showing the clustering analysis results of the scRNA-seq data for different ages. **(E,F)** Heatmaps showing the top 5 DEGs for each cell type at different ages.

Supplementary Figure 2 Volcano plots showing the DEGs between IHCs and OHCs at P14 and P28; the top 20 DEGs for IHC/OHC were labeled.

Supplementary Figure 3 The scaled expression level of top 100 DEGs between different ages along with the pseudotime.

Supplementary Figure 4 GSEA biological process activated and suppressed in DEGs from comparing P14 HCs vs. P7 HCs, and P28 HCs vs. P14 HCs.

Supplementary Figure 5 Violin plots showing the expression levels of *Miat* and *Pcp4* at E14, E16, P1, and P7. Figures were constructed from gEAR (https://umgear.org/) by using Kolla et al.’s data.
