# Supplementary figures and images for: Profiling mouse cochlear cell maturation using 10× Genomics single-cell transcriptomics
Source: Front Cell Neurosci. 2022 Aug 18;16:962106. doi: 10.3389/fncel.2022.962106 (PMC9434313; doi:10.3389/fncel.2022.962106)

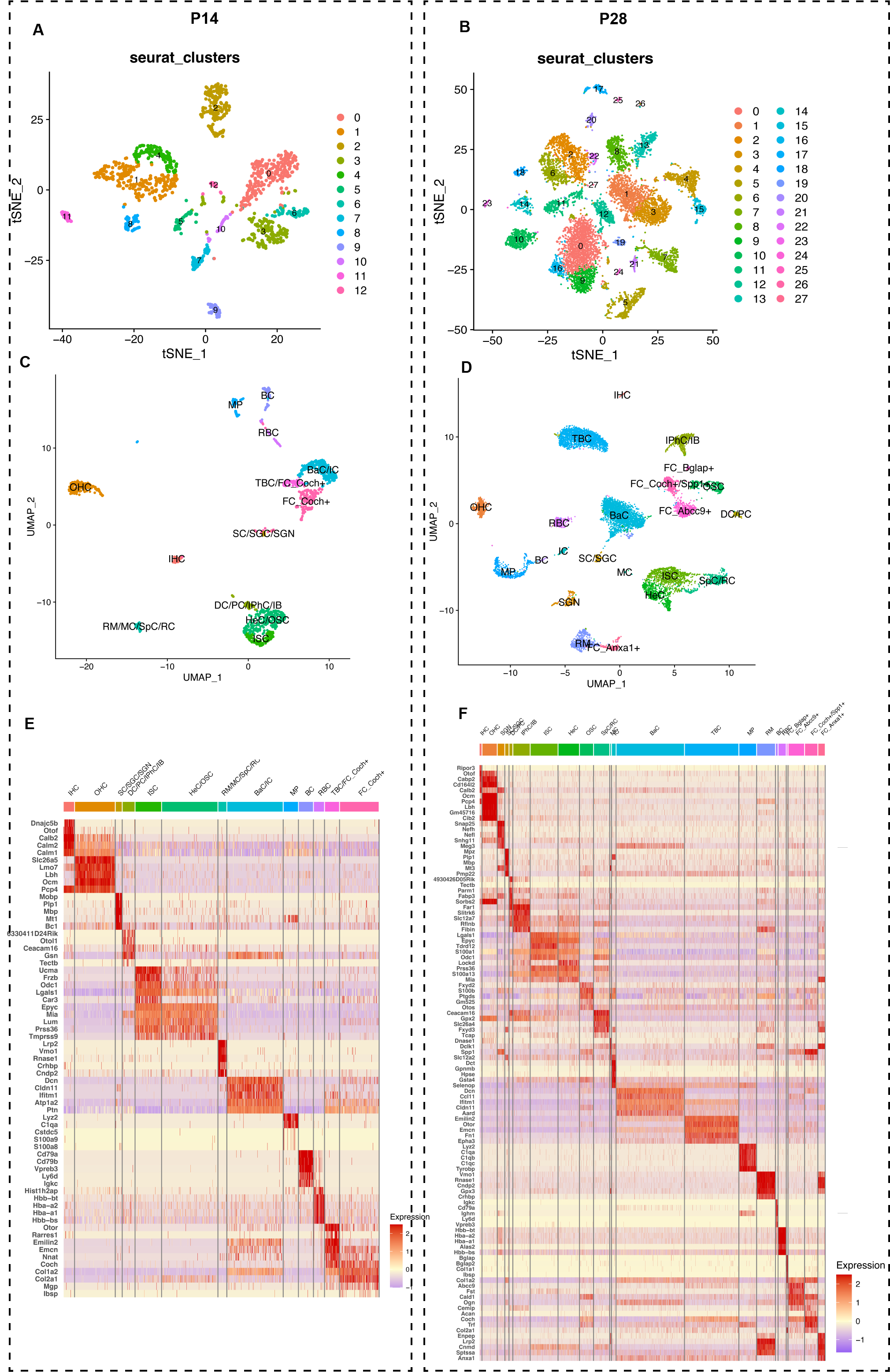

Supplement: Supplementary Figure 1 — (A–D) tSNE and UMAP plots showing the clustering analysis results of the scRNA-seq data for different ages. (E,F) Heatmaps showing the top 5 DEGs for each cell type at different ages. [file Image_1.JPEG]

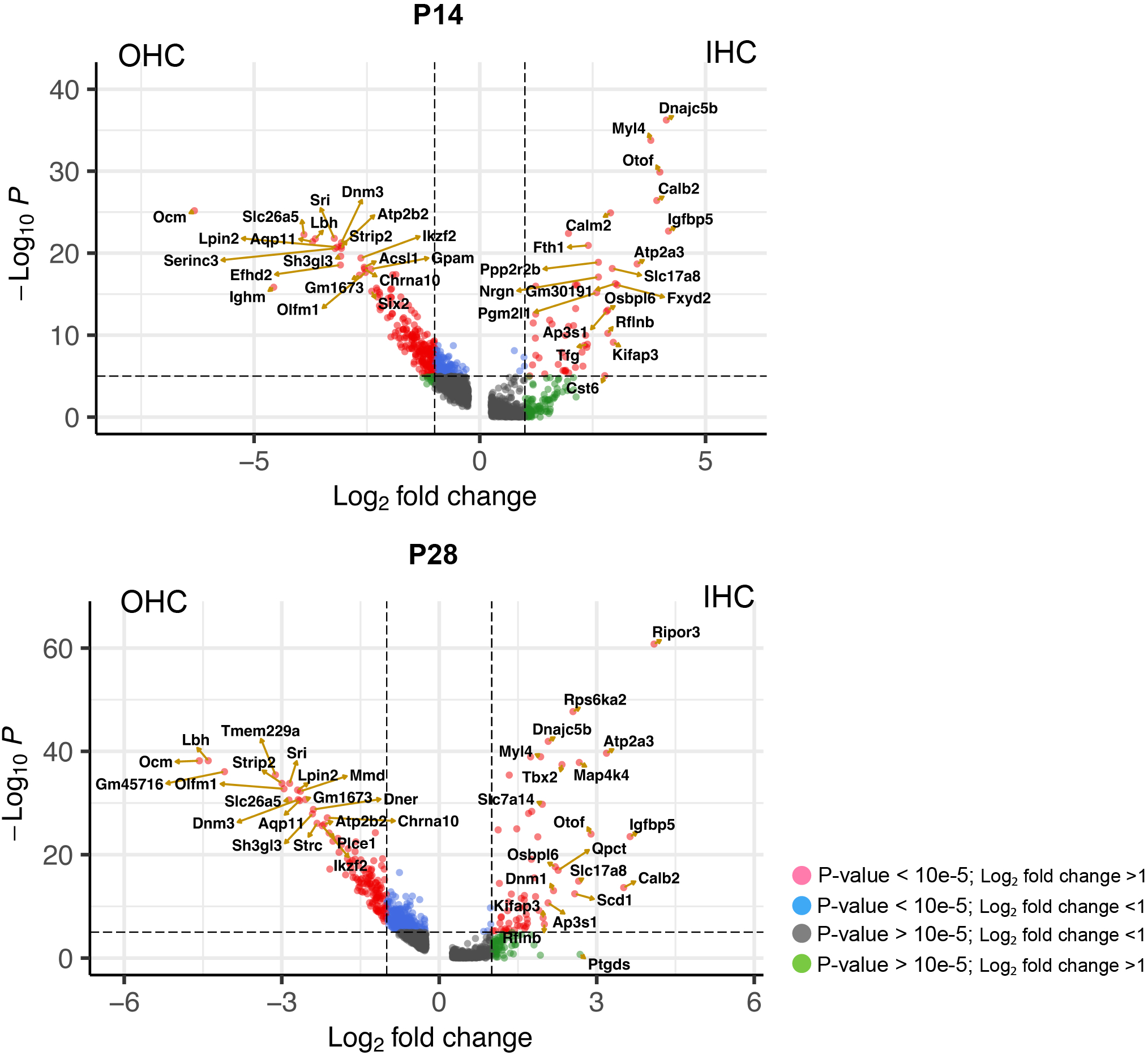

Supplement: Supplementary Figure 2 — Volcano plots showing the DEGs between IHCs and OHCs at P14 and P28; the top 20 DEGs for IHC/OHC were labeled. [file Image_2.JPEG]

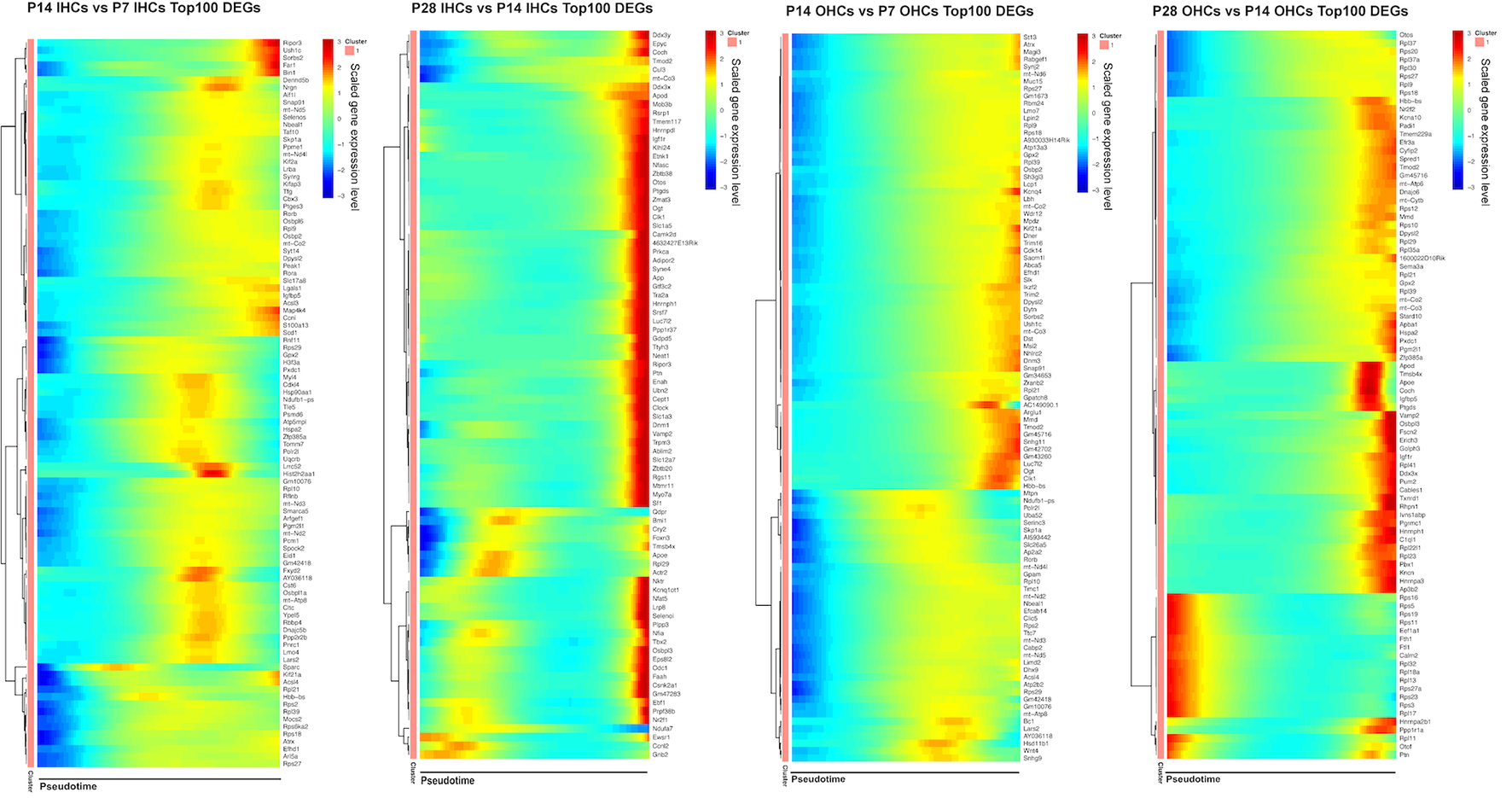

Supplement: Supplementary Figure 3 — The scaled expression level of top 100 DEGs between different ages along with the pseudotime. [file Image_3.TIF]

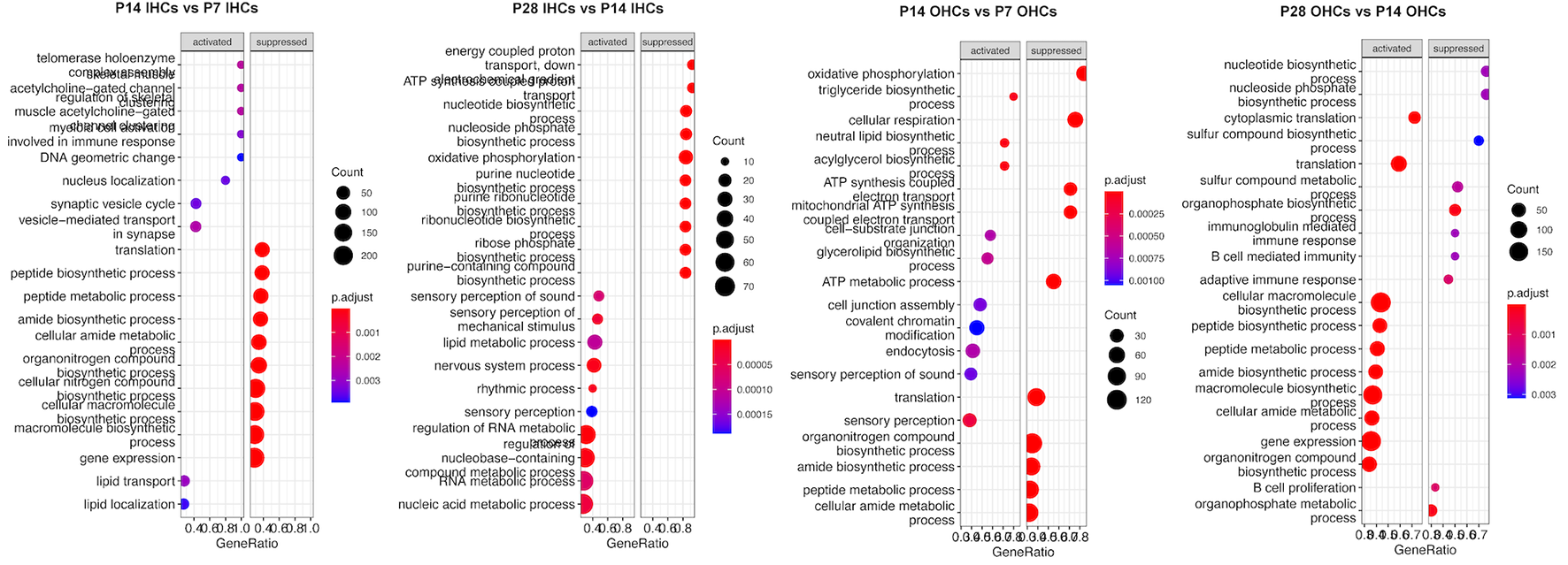

Supplement: Supplementary Figure 4 — GSEA biological process activated and suppressed in DEGs from comparing P14 HCs vs. P7 HCs, and P28 HCs vs. P14 HCs. [file Image_4.TIF]

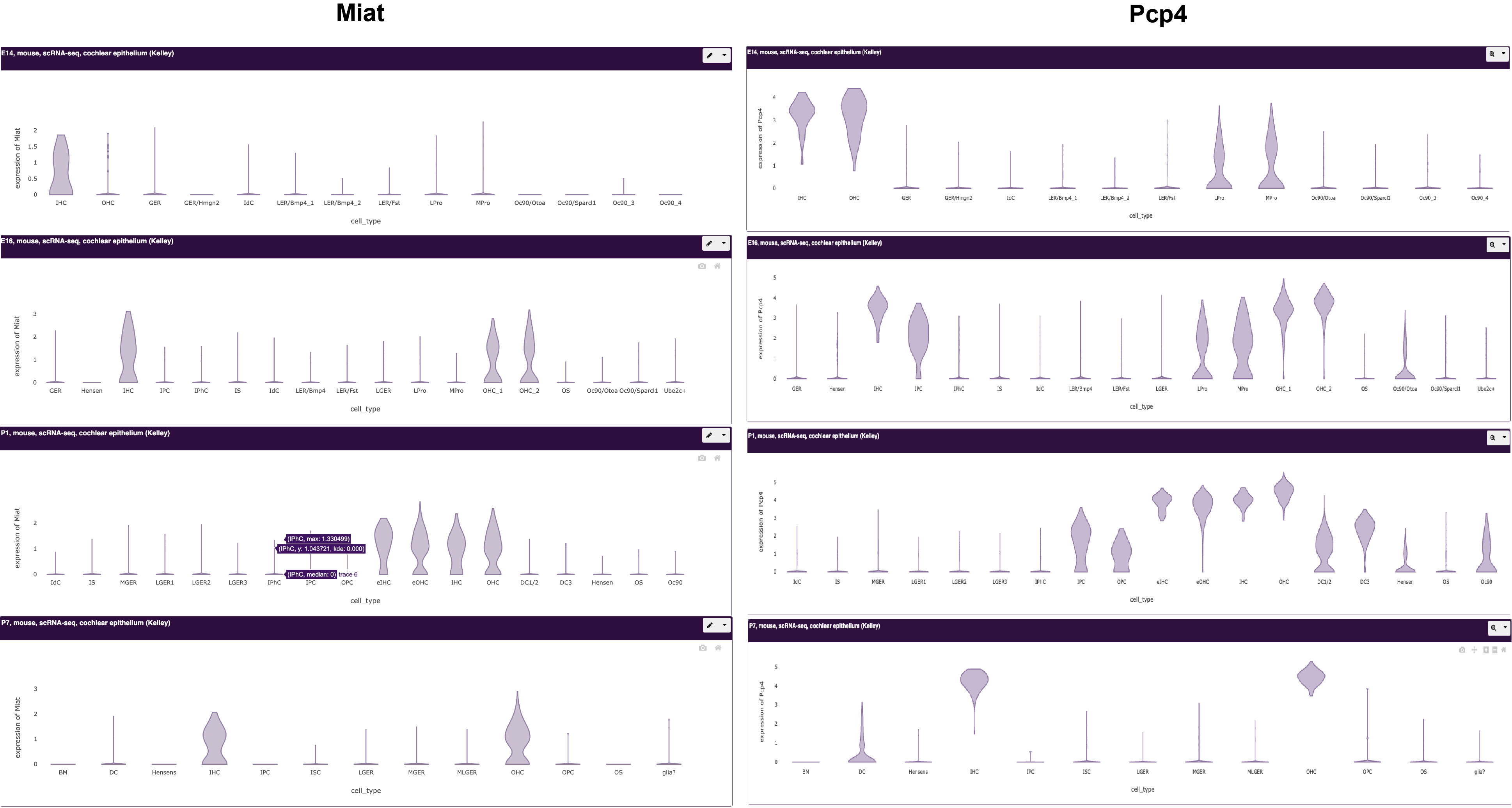

Supplement: Supplementary Figure 5 — Violin plots showing the expression levels of Miat and Pcp4 at E14, E16, P1, and P7. Figures were constructed from gEAR (https://umgear.org/) by using Kolla et al.’s data. [file Image_5.JPEG]
